# C-arm CT scanning combined with simple laser device-assisted puncture therapy for cerebellar hemorrhage

**DOI:** 10.3389/fsurg.2024.1421517

**Published:** 2024-10-24

**Authors:** Yang Chen, Chenglong Li, Qingbo Wang, Zefu Li

**Affiliations:** Department of Neurosurgery, Binzhou Medical University Hospital, Binzhou, Shandong, China

**Keywords:** laser-assisted navigation, C-arm CT scanning, minimally invasive puncture, cerebellar hemorrhage, precision medicine

## Abstract

**Background:**

Cerebellar hemorrhage is a severe cerebrovascular disease. The small posterior fossa space can cause compression of surrounding brain tissue with even a small amount of bleeding, leading to increased intracranial pressure, disruption of blood supply to surrounding brain tissue, and exacerbation of brain function damage. The most common surgical approach currently is craniotomy for hematoma evacuation, inevitably causing damage to surrounding nerves and blood vessels. In this study, we introduced C-arm CT scanning combined with simple laser device technology to assist in puncture drainage for cerebellar hemorrhage, aiming to improve the accuracy of surgery and maximize the protection of patients’ brain function, providing a physiological basis for better clinical prognosis.

**Materials and methods:**

From January 2023 to February 2024, a total of 8 patients (6 males, 2 females) with cerebellar hemorrhage underwent C-arm CT-assisted puncture therapy combined with a simple laser device at the affiliated hospital of Binzhou Medical University. Statistical analysis was performed on operation time, number of punctures, impact on important structures and vessels, postoperative hematoma clearance, complications, and neurological function recovery.

**Results:**

All 8 patients underwent the surgery smoothly without causing damage to important structures or blood vessels. There was no rebleeding intraoperatively. Among the 8 patients, 5 were discharged smoothly, while 3 patients opted to discontinue treatment and requested discharge. At the 3-month follow-up, 3 patients showed no ataxia, while 2 patients had impaired cerebellar motor function.

**Conclusion:**

C-arm CT scanning combined with a simple laser device technology can accurately locate the position of the hematoma, effectively avoid important structures and vessels, reduce damage to surrounding normal brain tissue, and maximize the protection of normal brain tissue function. Real-time navigation and dynamic adjustments during surgery allow immediate access to imaging data postoperatively. It also has the advantages of being minimally invasive, highly precise, easy to operate, and short operation time, demonstrating high practicality and feasibility.

## Introduction

Cerebellar hemorrhage is a severe cerebrovascular disease, accounting for about 10% of spontaneous intracerebral hemorrhages, often leading to serious neurological dysfunction and even life-threatening conditions (1). Although cerebellar hemorrhage is relatively rare compared to ischemic strokes, its high mortality and disability rates are concerning. The small posterior fossa space can cause compression of surrounding brain tissue with even a small amount of bleeding, leading to increased intracranial pressure, disruption of blood supply to surrounding brain tissue, and exacerbation of brain function damage. This activates the release of immune cells and inflammatory mediators, disrupting neurotransmitter balance (2), resulting in various clinical symptoms including headaches, nausea, vomiting, consciousness disorders, increased muscle tone, and motor disorders. Severe bleeding can even rupture into the ventricular system and/or cause compression of the fourth ventricle, leading to obstructive hydrocephalus. Compression of the brainstem causes a syndrome of disturbed vital signs, including damage to the reticular formation and areas controlling respiration and heart rate, leading to coma, respiratory failure, and unstable blood pressure, posing a serious threat to life (3, 4). Early and effective hematoma evacuation to reduce hematoma pressure, minimize immune activation and inflammatory mediator release caused by bleeding, and maximize brain function protection is essential for improving patient outcomes (5).

The most common surgical approach currently is posterior fossa craniotomy for hematoma evacuation, which is one of the most common surgical approaches for cerebellar hemorrhage (6, 7). Surgeons open the patient's scalp and skull, then remove or reduce the hematoma compressing the cerebellum, inevitably causing damage to surrounding normal brain tissue. During the craniotomy, how to avoid damage to the transverse and sigmoid sinuses, closure of the posterior neck muscles, and postoperative bleeding and edema in the small posterior fossa, which can have more serious consequences than supratentorial surgery, all need to be fully evaluated by neurosurgeons (8). Minimally invasive puncture hematoma can solve the above problems well, but how to accurately locate the location of hematoma is worth considering (9).Some scholars have carried out a study on the treatment of spontaneous cerebellar hemorrhage with severe brain stem dysfunction by bedside positioning minimally invasive puncture, and achieved good results (10). We describe the treatment plan for cerebellar hemorrhage using C-arm CT scanning combined with a simple laser device-assisted puncture therapy, aiming to provide new treatment options for cerebellar hemorrhage, improve the accuracy of surgery, maximize the protection of patients’ brain function, reduce postoperative complications, and achieve better clinical prognosis (11).

## Materials and methods

This study included 8 patients who underwent C-arm CT-assisted puncture therapy combined with a simple laser device for cerebellar hemorrhage from January 2023 to February 2024 at Binzhou Medical University Hospital (Table 1). The new technology of C-arm CT-assisted puncture therapy combined with a simple laser device for cerebellar hemorrhage was reviewed and approved by the ethics committee of Binzhou Medical University hospital, and informed consent was obtained from the patients’ families. Inclusion criteria: diagnosed with cerebellar hemorrhage, confirmed by computed tomography scanning to have cerebellar hemorrhage, (1) relatively concentrated hematoma, hematoma larger than 10 mL; (2) accompanied by progressive neurological dysfunction; (3) compression of the brainstem causing unstable vital signs; (4) compression of the fourth ventricle leading to obstructive hydrocephalus. Exclusion criteria: (1) intracerebral hemorrhage due to secondary factors such as arteriovenous malformations, cerebral aneurysms, tumor strokes, etc.; (2) caused by head trauma; (3) intracranial hypertension without obvious clinical symptoms and imaging findings; (4) multiple intracerebral hemorrhages; (5) severe visceral diseases or coagulation disorders; (6) unstable blood pressure, heart rate; (7) patients with brain herniation.

**Table 1:** Preoperative basic data.

Case No	Age (years)	Sex (F/M)	Admission GCS	Dyskinesia (Y/N)	Volume of hematoma (mL)	Time from onset to operation (h)
1	74	F	11	Y	20.3	7
2	70	M	12	N	28.5	25
3	86	M	13	Y	12.8	12
4	87	M	12	N	21.7	5
5	89	F	9	Y	22.7	30
6	65	M	6	Y	30.2	5
7	73	M	14	Y	19.2	18
8	77	M	14	Y	18.1	24

### Equipment

**Figure 1:**
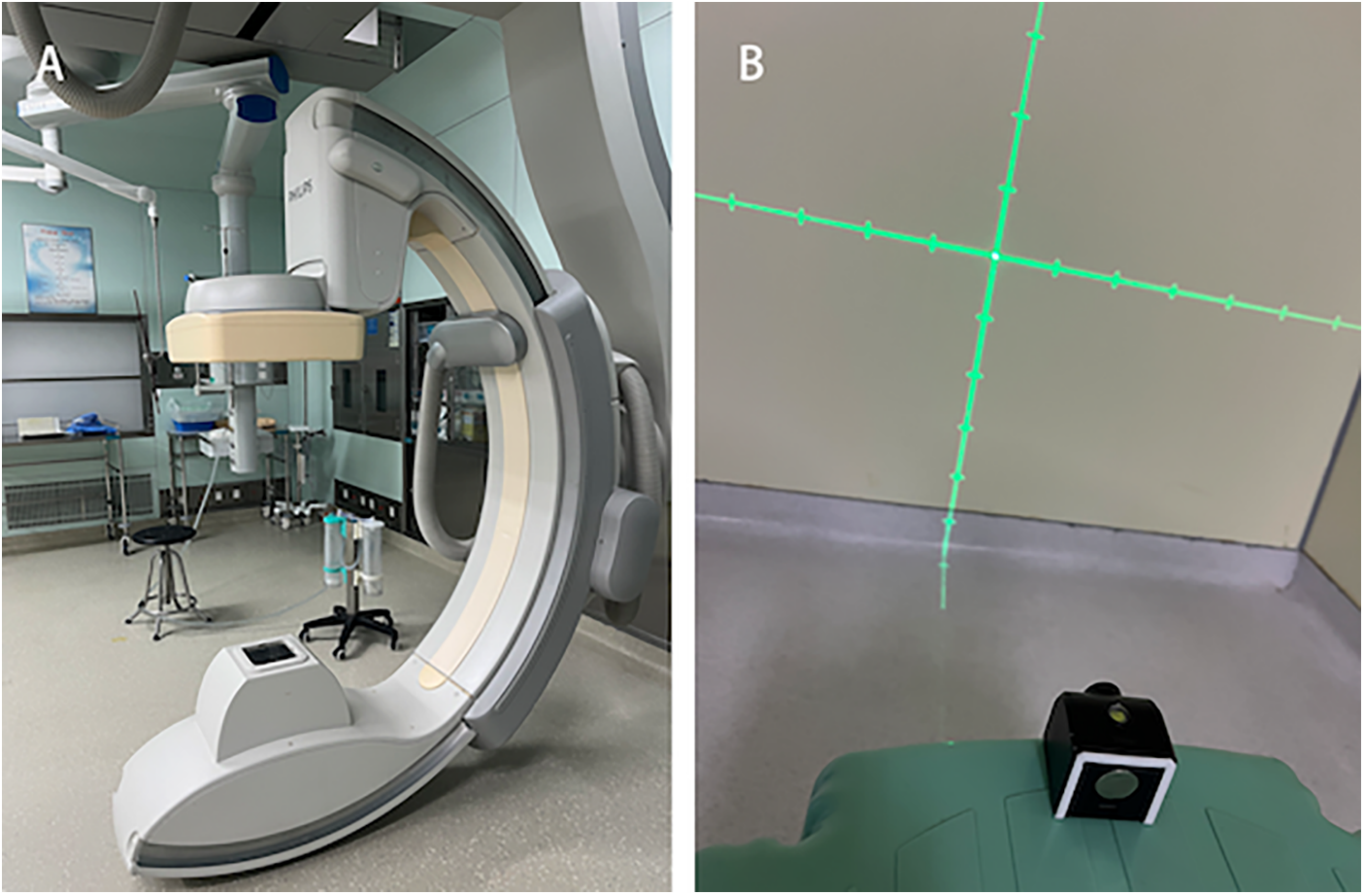
Equipment. **(A)** C-arm CT equipment (UNIQ FD20, Philips Healthcare, Netherlands). **(B)** Laser emitter.

1. C-arm CT equipment (UNIQ FD20, Philips Healthcare, Netherlands) - used for digital subtraction angiography (DSA) and computed tomography (CT), providing high-resolution vascular imaging and three-dimensional anatomical information for preoperative planning and intraoperative navigation Figure 1A.
2. Laser emitter (Zhongna NX-9575-675, Zhongshan Zhongna Electronics Co., Ltd.) - used for positioning and navigation during surgery, providing accurate optical guidance to help determine puncture points and hematoma locations Figure 1B.

### Surgical procedure

#### Puncture site marking

**Figure 2:**
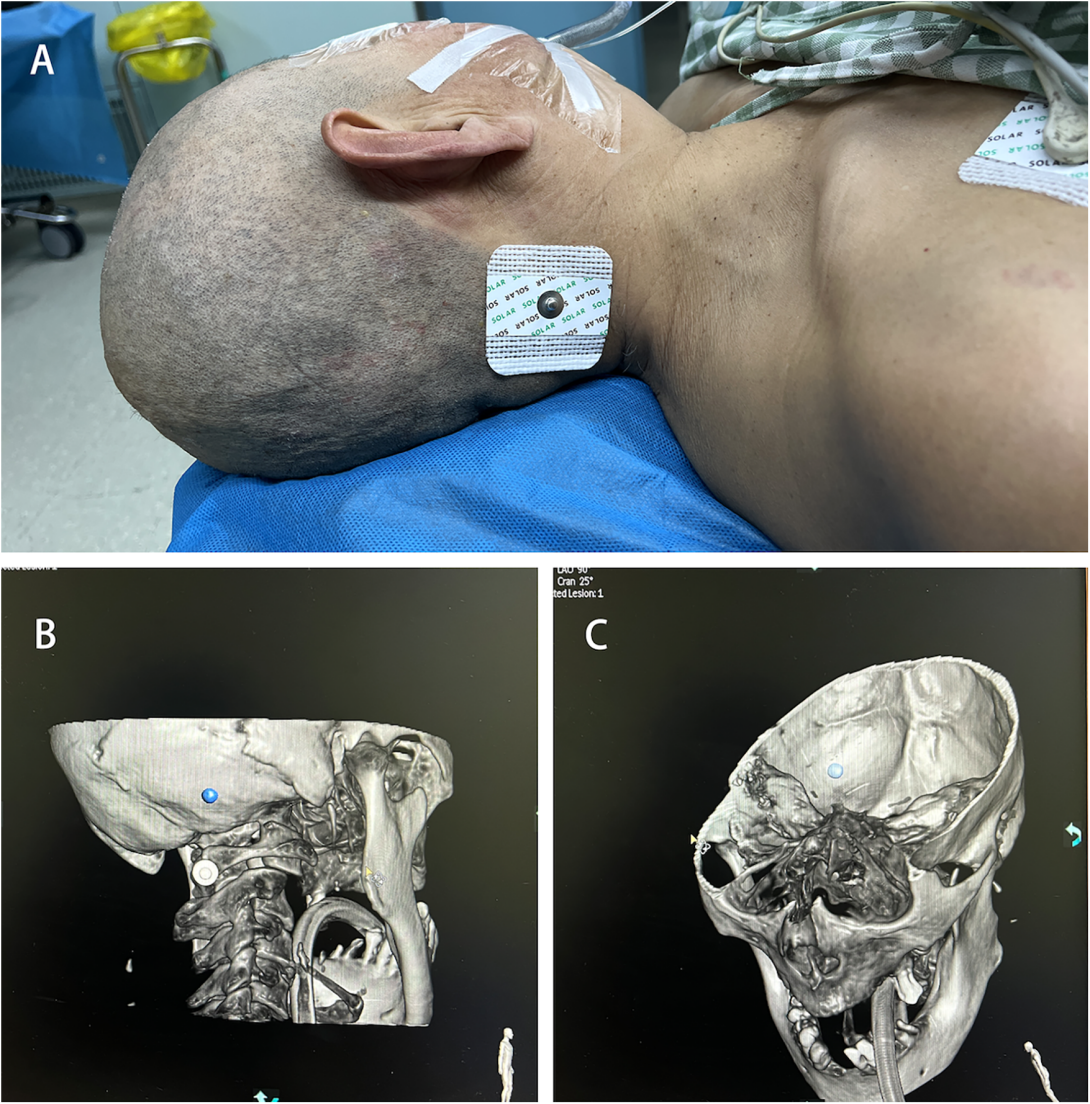
Puncture site marking. **(A)** Pre-puncture point. **(B)** C-arm CT scan marking bone hole position. **(C)** Avoidance of transverse sinus and sigmoid sinus groove.

Position the patient's head laterally to fully expose the occipital area on the side of the hematoma. Attach an electrode pad approximately 2–3 cm beside the mastoid process and about 2 cm below the occipital protuberance as the pre-puncture point Figure 2A. (Due to the limitation of the C-arm CT rotation angle, using this position as the pre-puncture point maximizes the chance of maintaining an unrestricted puncture angle and serves as a reference point for more accurate adjustment of the bone aperture position.) Perform a C-arm CT scan to identify the locations of the transverse sinus and sigmoid sinus groove, avoiding the transverse sinus, sigmoid sinus, and critical functional areas. Determine the bone aperture location Figures 2B,C, and confirm the skin incision site once again.

#### Surgery

After anesthesia and routine disinfection, a skin incision of approximately 2.5 cm is made at the predetermined puncture site, and a bone hole with a diameter of 0.5 cm is created using a bone drill.

**Figure 3:**
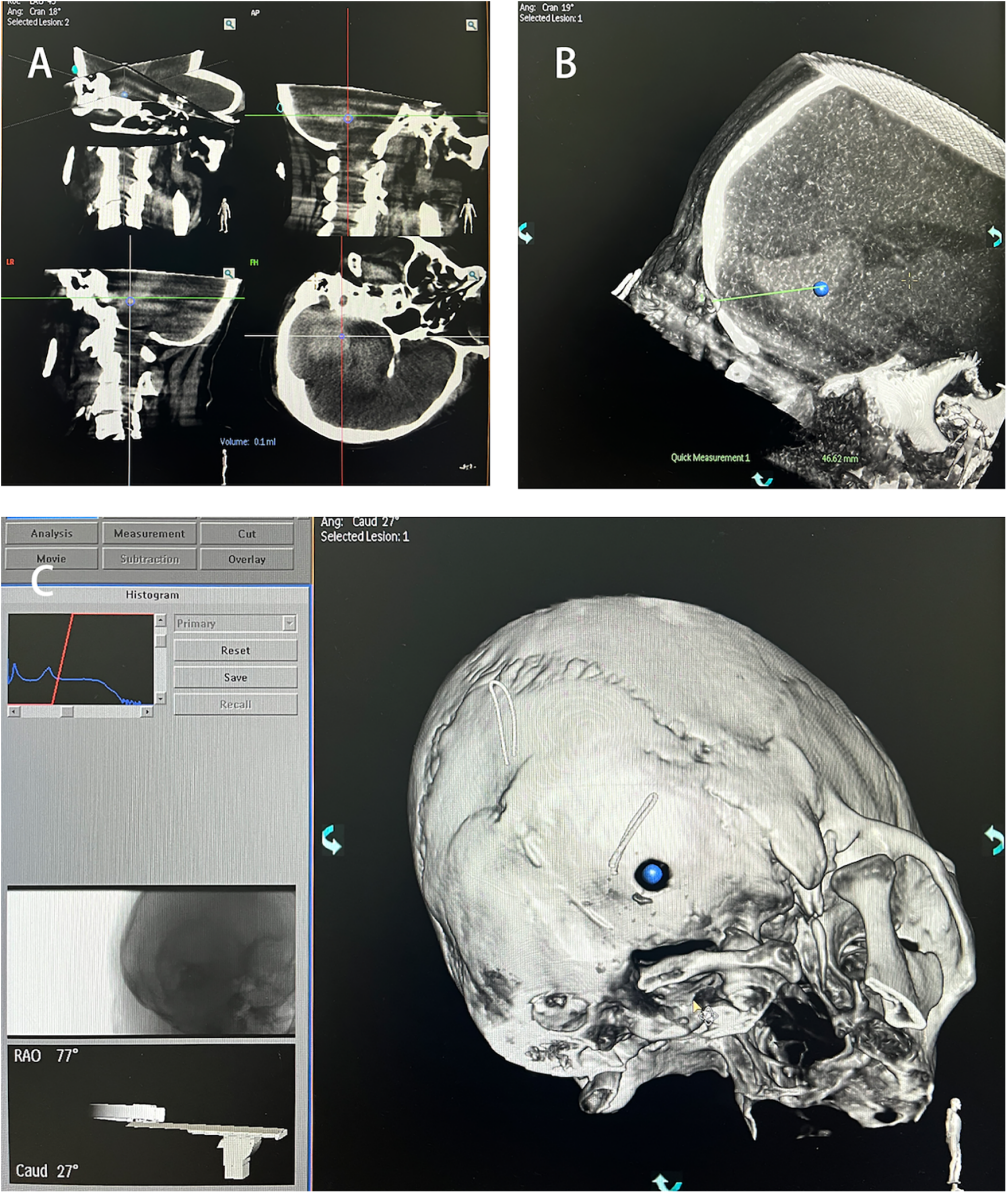
Puncture target identification. **(A)** Hematoma target marking. **(B)** Distance from bone orifice to hematoma target. **(C)** Spatial angle of puncture direction.

Perform another C-arm CT scan and use the software provided by the machine for 3D reconstruction to determine the maximum axial, coronal, and sagittal planes of the hematoma. Use the center of the maximum horizontal plane of the hematoma as the puncture target point Figure 3A. Rotate the 3D image to align the bone aperture with the determined puncture target point Figure 3C. At this point, the machine will display the spatial angle corresponding to the puncture direction. Record the working angle from the reference image, then slice the 3D image and measure the puncture depth Figure 3B. The straight-line distance from the percutaneous puncture point to the target point is taken as the puncture depth.

#### Puncture

**Figure 4:**
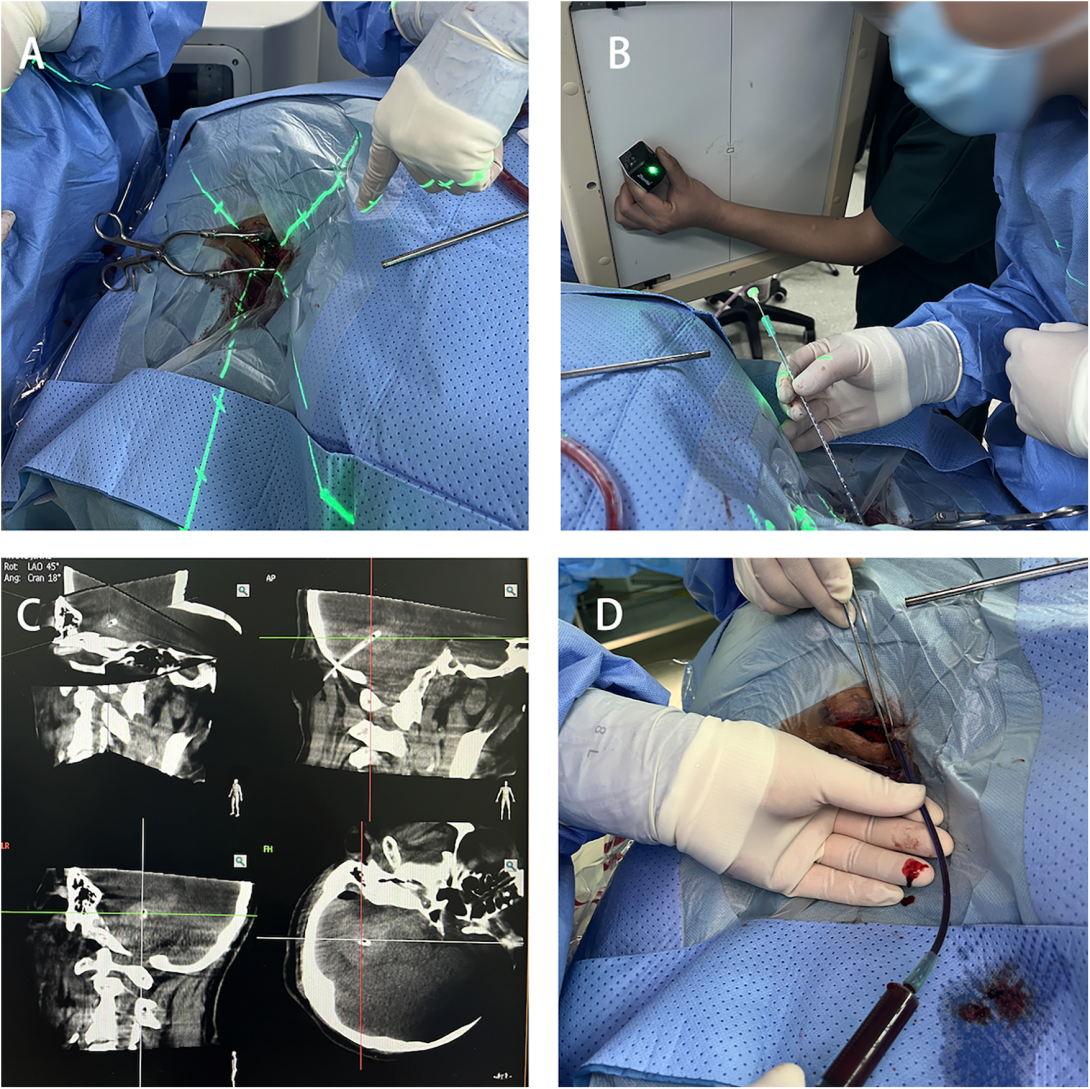
Hematoma puncture. **(A)** Laser crosshair aligning with the bone hole position. **(B)** Puncture drainage tube positioned at the center of the laser crosshair. **(C)** C-arm CT scan confirming the catheter position. **(D)** Aspiration of part of the hematoma for decompression.

Using the 3D-APC function in the automatic positioning control module of the C-arm CT, move the C-arm to the angle displayed in the 3D reference image. Fix the laser emitter at the center of the CT emitter plate, aligning the crosshair of the laser emitter with the bone Figure 4A. Select a 12F external ventricular drainage catheter and use the linear motion of the light to align the tail end of the needle core of the puncture catheter with the crosshair Figure 4B. Slowly advance the puncture needle along the laser emission direction until it reaches the designated puncture depth, then stop the insertion and temporarily fix the drainage catheter. Perform another C-arm CT scan to verify the puncture position and the trajectory of the drainage catheter Figure 4C. After confirming the catheter has reached the predetermined point, use a 10 mL syringe to slowly aspirate the hematoma (approximately one-third of the hematoma volume) to reduce the pressure on the surrounding brain tissue Figure 4D. Finally, secure the drainage catheter, suture the skin, and conclude the procedure.

### Postoperative follow-up

A postoperative cranial CT scan performed within 24 h showed no intracranial hemorrhage. Urokinase solution (50,000 IU in 5 mL of saline) was injected into the hematoma cavity through the drainage catheter. The catheter was clamped for 2 h and then reopened. Urokinase was injected twice daily, with a total of two consecutive injections. The procedure was conducted under strict aseptic conditions. A follow-up cranial CT scan was performed one day later to assess the intracranial condition, and the drainage catheter was removed when appropriate (12).

## Results

**Figure 5:**
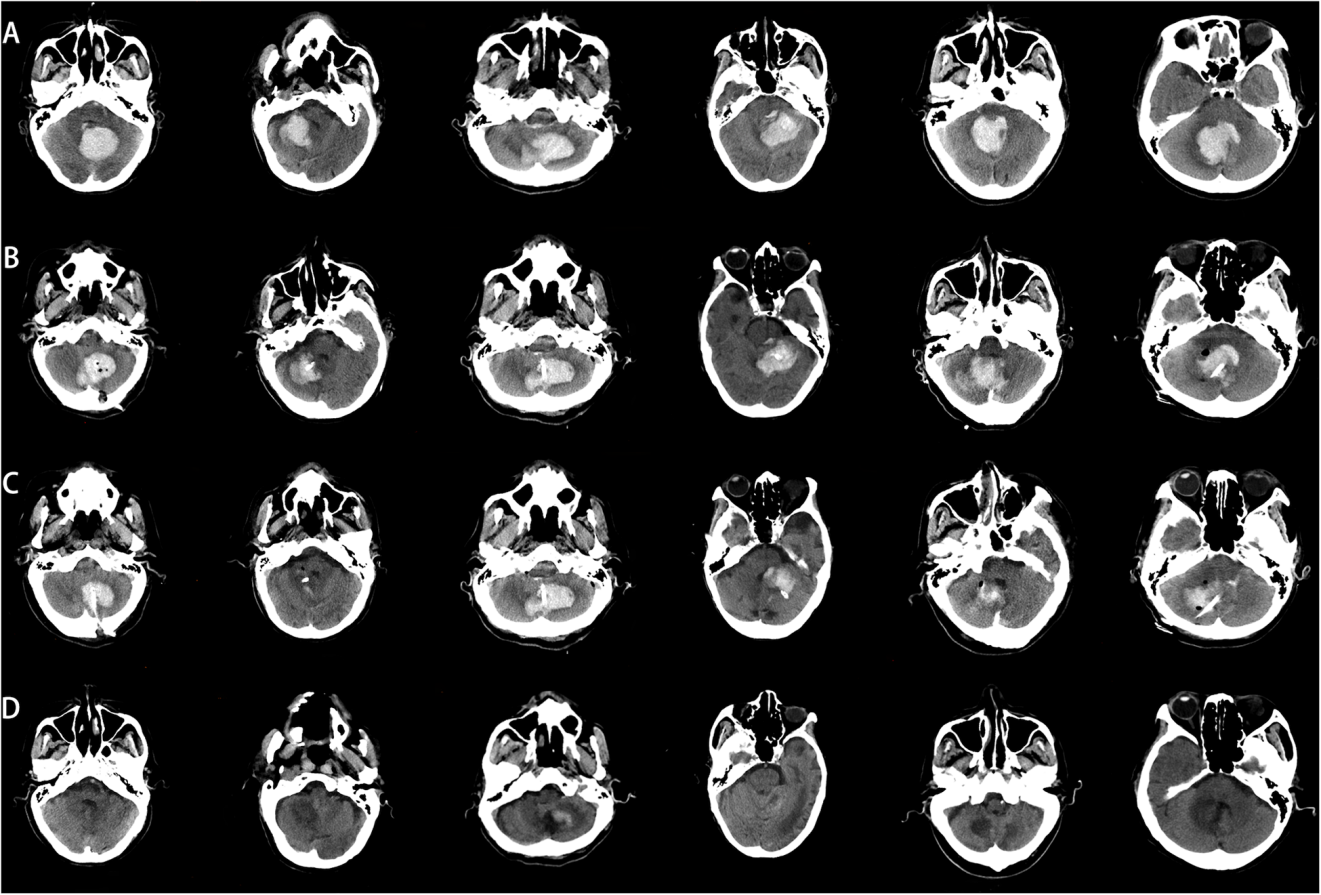
Comparison of preoperative and postoperative CT images. **(A)** Preoperative CT image, **(B)** CT image 24 h postoperatively, **(C)** CT image 3 days postoperatively. **(D)** CT image 15 days postoperatively.

All patients successfully completed the surgery, with no intraoperative deaths or postoperative rebleeding. Postoperative CT results showed that the tip of the puncture catheter was located within the hematoma cavity, the hematoma volume was reduced compared to the preoperative state, and the compression of the fourth ventricle by the hematoma was alleviated. The combined effect of hematoma drainage and urokinase injection was satisfactory. Follow-up cranial CT scans were performed 24 h, 3 days, and 15 days postoperatively Figure 5. Among the 8 patients, 5 were discharged in good condition, while 3 chose to discontinue further treatment and requested discharge Tables 2, 3.

**Table 2:** Surgical information and postoperative outcomes.

Case no	Operation time (min)	Important vein injury (Y/N)	First successful puncture (Y/N)	Cerebellar tonsillar herniation (Y/N)	Postoperative hematoma volume (mL)
1	55	N	Y	N	15.3
2	75	N	Y	N	20.3
3	95	N	N	N	8.1
4	85	N	Y	N	15.1
5	65	N	Y	N	14.3
6	50	N	Y	N	18.7
7	115	N	Y	N	10.4
8	135	N	Y	N	8.9

**Table 3:** Postoperative patient outcomes.

Case no	Intracranial infection (Y/N)	Hydrocephalus (Y/N)	Outcome	Discharge GCS	Ataxic dyskinesia 3 months after surgery (Y/N)
1	N	N	Recover	13	N
2	Y	N	Abandon	12	–
3	Y	N	Recover	14	N
4	N	N	Abandon	9	–
5	N	N	Abandon	7	–
6	N	N	Recover	5	Y
7	N	N	Recover	14	Y
8	N	N	Recover	15	N

## Discussion

The cerebellum is located in the posterior fossa of the skull, separated from the occipital lobes of the cerebrum by the tentorium cerebelli. It is connected to the brainstem via three pairs of peduncles. The cerebellum plays a crucial role in motor coordination, maintaining balance, regulating muscle tone, and coordinating voluntary movements. Cerebellar hemorrhage can lead to life-threatening complications such as brainstem compression, obstructive hydrocephalus, and neurological deficits due to compression of surrounding brain tissue (13).

The current traditional surgical approach involves removing the skull, extracting blood clots from the cerebellum (6), inevitably causing damage to surrounding normal brain tissue. The cerebellum receives a large amount of sensory information related to movement and information from the cerebral cortex related to motor centers. Its efferent fibers directly or indirectly affect the functions of the spinal cord, brainstem, and cerebral cortex. Therefore, the cerebellum is an important center for regulating movement in the central nervous system. This is manifested in maintaining body balance, regulating muscle tone, and coordinating muscle movements (coordination) (14). Secondary damage caused by clearing hematomas can result in patients experiencing balance disorders, coordination disorders, and ataxia. Therefore, the key focus is on effectively clearing hematomas, reducing their impact on surrounding normal tissues, and protecting cerebellar tissue to the maximum extent possible (9).

With advancements in modern imaging technology, techniques such as stereotactic guidance, intraoperative navigation, and robotic assistance have been introduced to neurosurgical procedures, providing technical support for minimally invasive puncture therapy for intracerebral hemorrhage (15). However, these techniques still have limitations (16, 17).

Our center has developed a technique using C-arm CT scanning combined with a simple laser device for puncture therapy of intracerebral hemorrhage, which has been successfully applied in supratentorial hematoma drainage (18, 19). This technique offers several advantages, including minimal invasiveness, real-time navigation, dynamic adjustments, and immediate postoperative imaging evaluation, thereby improving the accuracy and success rate of surgery while reducing the risk of complications.

In our study, all patients had cerebellar hemorrhage with hematomas larger than 10 mL (20). After excluding vascular malformations, we communicated thoroughly with the patients’ families and used C-arm CT scanning combined with a simple laser device to assist in puncture drainage. Early release of hematoma pressure is crucial for improving patient prognosis. All patients underwent successful surgery without intraoperative deaths, and postoperative CT scans confirmed the proper placement of the drainage tubes within the hematoma cavity (Figure 5).

Using a simple laser device combined with C-arm CT scanning for assisted cerebellar hematoma puncture drainage has the following advantages: (1) Reduced surgical trauma compared to traditional craniotomy for hematoma removal, minimizing damage to surrounding normal brain tissue and blood vessels, thus preserving cerebellar function to the maximum extent. Additionally, craniotomy procedures are time-consuming and increase the risks of anesthesia and infection, enhancing patient quality of life. (2) Flexibility with real-time navigation and dynamic adjustments during surgery, allowing for real-time adjustments to unpredictable situations, ensuring a higher success rate in the first puncture and reducing the number of punctures. (3) Effective avoidance of important structures and blood vessels by adjusting the drainage tube position based on the scan results, minimizing puncture damage and achieving the goal of precision medicine. (4) Immediate postoperative imaging to confirm the accuracy of the puncture. (5) Simple and convenient operation with low learning costs.

In summary, utilizing the technology of combining C-arm CT scanning with a simple laser device enables precise localization of the hematoma, effective avoidance of important structures and blood vessels, real-time navigation, and dynamic adjustments during surgery. It also offers advantages such as reduced trauma, high precision, shorter operation time, and immediate postoperative imaging confirmation of puncture accuracy, demonstrating high practicality and accessibility.

## Data Availability

The original contributions presented in the study are included in the article/Supplementary Material, further inquiries can be directed to the corresponding author.
